# sncRNA signatures underlying HIV-1 restriction in elite controllers

**DOI:** 10.3389/fcimb.2026.1926123

**Published:** 2026-10-20

**Authors:** Rubén Ayala-Suárez, Erick De la Torre Tarazona, Mercedes Bermejo, Manel E. Bargalló, María José Maleno, Mayte Coiras, Lorna Leal, Felipe García, Montserrat Plana, Javier García-Pérez, José Alcamí, Francisco Díez-Fuertes

**Affiliations:** 1 AIDS Immunopathology Unit, Centro Nacional de Microbiología, Instituto de Salud Carlos III (ISCIII), Madrid, Spain; 2 Infectious Diseases Department, Hospital Universitario Ramón y Cajal, Instituto Ramón y Cajal de Investigación Sanitaria (IRYCIS), Madrid, Spain; 3 Centro de Investigación Biomédica en Red de Enfermedades Infecciosas (CIBERINFEC), Instituto de Salud Carlos III, Madrid, Spain; 4 AIDS and HIV Research Group, Institut d´Investigacions August Pi i Sunyer (IDIBAPS) Hospital Clinic, University of Barcelona, Barcelona, Spain; 5 Immunopathology and Viral Reservoir Unit, Centro Nacional de Microbiología, Instituto de Salud Carlos III (ISCIII), Madrid, Spain; 6 Infectious Diseases Service, Hospital Clínic, University of Barcelona, Barcelona, Spain

**Keywords:** CD4 T cells, gene regulation, HIV control, HIV-1 infection, miR-125a-5p, miR-99b-5p, miRNA, sncRNA

## Abstract

**Introduction:**

HIV controllers maintain low viral loads without therapy, offering a natural model for a functional cure. The regulatory role of the small non-coding RNA (sncRNA) transcriptome in CD4+ T cells has been scarcely explored. Our study aimed to characterize the sncRNA and coding RNA landscape in CD4+ T cells from HIV controllers and to assess the potential antiviral activity of key non-coding RNAs.

**Methods:**

We performed sncRNA-Seq and RNA-Seq on CD4+ T cells from elite controllers (ECs, n=10), viremic controllers (n=6), and untreated (n=8) and treated (n=9) non-controllers. Differential expression analysis, miRNA-mRNA correlation, interaction mapping, and functional annotation were conducted. To assess antiviral activity, inhibitors for candidate miRNAs were transfected into CEM-NKR.CCR5 cells, which were then infected with a replication-competent HIV-1 clone.

**Results:**

The sncRNA compartment of elite controllers was distinct, including overexpression of specific transfer RNA (tRNA)-Lys isoacceptors and a cluster of five PIWI-interacting RNAs (piRNA) associated with tRNA-Lys genomic repeats. Noteworthy, miR-99b-5p and miR-125a-5p were significantly downregulated in elite controllers compared to both non-controller groups. Concurrently, the coding transcriptome of elite controllers displayed an enrichment in p53 signaling, inflammatory response, cell activation, and oxidative stress. Several genes upregulated in these processes (e.g., *THBS1*, *DPM2*) were identified as validated targets of miR-99b-5p and miR-125a-5p. Finally, *in vitro* experiments showed that miR-99b-5p and miR-125a-5p inhibition, individually or in combination, reduced viral replication by 17.6%, 16.3%, and 21.4%, respectively.

**Discussion:**

Elite controllers exhibit a distinctive sncRNA signature in CD4+ T cells. The coordinated downregulation of miR-99b-5p and miR-125a-5p may contribute to a restrictive cellular environment and reducing viral replication, revealing a novel layer of post-transcriptional regulation in natural HIV control. These findings underscore these miRNAs and their potentially regulated counterparts as potential targets for future therapeutic strategies.

## Graphical Abstract

**Figure:**
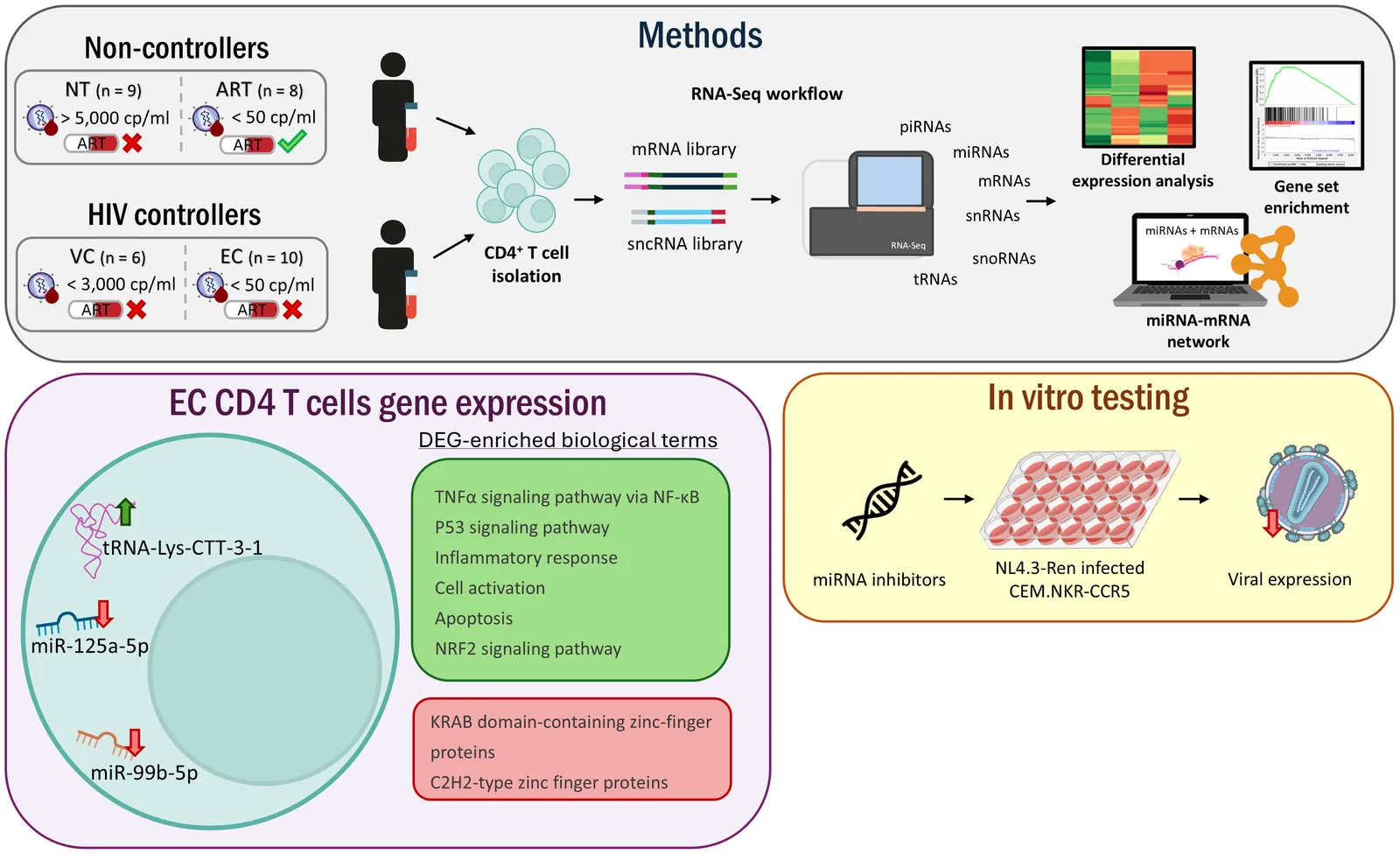
Elite controllers show a distinct CD4^+^ T cell small non-coding RNA profile, including altered tRNAs and downregulated miR-99b-5p and miR-125a-5p. These changes correlate with enhanced antiviral, inflammatory, and metabolic pathways. Inhibition of these miRNAs reduces HIV replication, suggesting they contribute to natural viral control and may represent therapeutic targets.

## 1 Introduction

The HIV/AIDS pandemic continues to pose a significant global health challenge. Even though antiretroviral therapy has transformed HIV infection into a manageable chronic condition, critical goals remain elusive, including the achievement of a preventive vaccine and a functional cure (Lichterfeld et al., 2022). A limited group of people with HIV (PWH), known as HIV controllers, are able to control HIV-1 replication in the absence of antiretroviral therapy. Elite controllers (EC) and viremic controllers (VC), differing on their ability to suppress viral load (Gurdasani et al., 2014), offer a valuable model for identifying mechanisms of viral control and targets for therapeutic strategies to achieve a cure for HIV infection.

HIV control is a multifactorial condition, mainly driven by a favorable genetic background and robust innate and adaptive immune responses (Navarrete-Muñoz et al., 2020; Mastrangelo et al., 2022). Features associated with HIV control include protective HLA alleles (e.g., -B*57 and -B*27), improved viral sensing by innate immune cells, enhanced NK cell and T-follicular helper functions, and potent HIV-specific CD8+ T cell responses (Pereyra et al., 2010; Calvet-Mirabent and Martín-Gayo, 2022; Salgado et al., 2024; Sánchez-Gaona et al., 2024). Transcriptomics studies have further displayed alterations in interferon signaling, T-cell activation, and apoptosis in HIV controllers (De La Torre-Tarazona et al., 2022). However, most research has focused on protein-coding genes, leaving the role of non-coding RNAs, poorly explored in this context.

Small non-coding RNAs (sncRNAs) are functional RNAs sized up to 200 nucleotides that do not encode proteins but regulate diverse cellular functions. Major families include microRNAs (miRNAs), transfer RNAs (tRNAs), PIWI-interacting RNAs (piRNAs), small nuclear RNA (snRNAs), and small nucleolar RNAs (snoRNAs). These molecules fine-tune gene expression through a wide diversity of functions, such as RNA interference (RNAi), mRNA splicing, RNA modification, and repression of transposable elements (Morris and Mattick, 2014; Wu et al., 2020). Given their pivotal regulatory role, dysregulation of sncRNAs has been implicated in numerous diseases, including cancer, autoimmune disorders, and infectious and metabolic diseases (Mori et al., 2018; Watson et al., 2019; Rosace et al., 2020). Among the sncRNAs, miRNAs are the best characterized; they participate in and guide the RNA-induced silencing complex (RISC) to target mRNAs, leading to translational repression or decay according to their complementarity. By dampening fluctuations in gene expression, miRNAs increase the robustness of gene expression regulatory networks and buffer cellular responses against perturbations such as infections (Berezikov, 2011; Ha and Kim, 2014; Gebert and MacRae, 2019).

Several studies have reported that miRNAs may influence on HIV infection by targeting HIV mRNAs or essential host cellular processes for viral replication, such as susceptibility to infection (Wang et al., 2009; Orecchini et al., 2014; Farberov et al., 2015; Quaranta et al., 2015; Okoye et al., 2021), replication (Farberov et al., 2015; Okoye et al., 2021), or the establishment and maintenance of latency (Detsika et al., 2012; Ruelas et al., 2015; Lu et al., 2017). Despite miRNA signatures have been associated to long-term non-progression and HIV control (Witwer et al., 2012; Reynoso et al., 2014; Ayala-Suárez et al., 2020), most descriptive studies have been conducted in heterogeneous cell populations (i.e., whole blood, plasma, or peripheral blood mononuclear cells [PBMCs]), rather than in CD4+ T cells, which are the primary target of HIV.

Thus, the role of sncRNA in CD4+ T cells during spontaneous HIV control is still poorly understood. To address this gap, we comprehensively characterized the sncRNA landscape in CD4+ T cells from PWH with HIV control phenotypes using transcriptomic approaches. Resting CD4+ T cells were studied to explore the physiological transcriptional and post-transcriptional state established *in vivo*. We focused particularly on miRNA-mediated regulatory networks and validated key findings using an *in vitro* infection model. Our study aimed to identify novel sncRNA signatures of HIV control that may open new avenues for therapeutic intervention.

## 2 Material and methods

### 2.1 Population of study

Samples were obtained from PWH under follow-up at the HIV Unit of the Hospital Clinic de Barcelona (Barcelona, Spain). The study was approved by the Clinical Research Ethics Committee of the Hospital Clínic de Barcelona (Registration 2012/7506). A total of 33 participants divided into 4 groups were enrolled: 1) elite controllers (EC, n= 10) defined as PWH who maintain viral load < 50 cp/ml for at least one year of follow-up in the absence of ART, 2) viremic controllers (VC, n = 6) with viral load below 4.000 cp/ml for at least one year of follow-up without ART administration, 3) non-controllers PWH ART naïve with viral loads above 5, 000 cp/ml for more than one year (NT, n= 8), and 4) non-controller PWH under ART with viral loads below 50 cp/ml for more than one year of follow-up (ART, n= 9). All participants included in the study had a CD4+ T cell count above 450 cells/mm^3^. At the time of sampling, EC and VC participants had been followed up for more than 5 years.

### 2.2 Sample collection and RNA isolation

PBMCs were isolated from whole blood by density gradient centrifugation using Ficoll-Hypaque reagent (Rafer, Spain). CD4+ T cells were then isolated using α-CD4 antibodies conjugated to AutoMACS^®^ magnetic microspheres (Miltenyi Biotec SL. Alarcón, Spain) in a MACS Separator instrument (Miltenyi Biotec SL) and then stored at -192 °C (Bargalló et al., 2017). Total RNA extraction was performed using the miRNeasy mini kit product (Qiagen Iberia SL, Barcelona, Spain) following the manufacturer’s instructions. RNA concentration and purity were measured on a NanoDrop™ One kit (Thermo Fisher Scientific, Waltham, MA, USA), and the quality was assessed using an RNA 6000 Nano chip on the Bioanalyzer 2100 kit (Agilent Technologies, Santa Clara, CA, USA).

### 2.3 Next-generation sequencing

For miRNA and other sncRNA, 500 ng of total RNA were used to synthesize libraries with the TruSeq Small RNA Library Prep kit (Illumina, San Diego, CA, USA) following the manufacturer’s instructions. Libraries were run in a 6% acrylamide gel TBE-Novex Invitrogen (Thermo Fisher Scientific) for 50 min at 165 V and bands of 140–160 bp were cut, eluted, and purified with the illustra™ GFX PCR DNA and Gel Band Purification kit (Merck, Darmstadt, Germany). TruSeq Stranded Total RNA kit (Illumina) was used to synthetize cDNA libraries from mRNA following the manufacturer’s protocol. Starting from 1 µg of total RNA from the same RNA extraction employed in sncRNA-seq, ribosomal RNA was removed with the RiboZero Plus Depletion kit supplement (Illumina) followed by RNA fragmentation to a size of 120–200 bp. After cDNA synthesis and ligation of adapters, libraries were then purified with AMPure XP magnetic beads (Beckman Coulter, Pasadena, CA, USA). The quality and size of the sncRNA and mRNA purified libraries were evaluated with a High Sensitivity DNA chip (Agilent Technologies). All libraries were sequenced on 75-cycle NextSeq 500/550 High Output Kit v2.5 cartridges (Illumina) on a NextSeq 500 machine (Illumina) at the Scientific-Technical Genomics Unit of the Instituto de Salud Carlos III (Madrid, Spain). All sncRNA libraries were sequenced on a single cartridge, whereas mRNA libraries were separated into four different cartridges distributing individuals equally according to their phenotypes to avoid batch effects and to obtain a sufficient sequencing depth. This multiplexing strategy allowed us to distribute the expected 400 million reads from the NextSeq 500/550 High Output kit cartridge among different libraries, achieving an approximate number of reads of 45 million reads per sample in the case of mRNA libraries and 6 million reads in the case of miRNAs.

### 2.4 Mapping and sequence annotation

Once the sncRNA reads were obtained, the FASTQC program (v 0.11.9, Babraham Bioinformatics, Cambridge) was used for the quality control of sequences. Filtration and trimming of reads were performed with cutadapt software (v3.4, Technical University Dortmund, Germany), retaining sequences between 15 and 40 bp after removal of adapters. The mirDeep2 software (v0.1.3, Max Delbruck Center, Berlin) was employed for miRNA alignment using reads between 18 and 30 bp using the miRNA database miRBase v22 as reference (Kozomara et al., 2019). The rest of the retained reads that did not align to miRNA sequences were aligned and annotated to other sncRNA using the modular COMPSRA software (v1.0.3, Harvard Medical School, Boston) with the default multi-mapping parameters, that employs STAR program to align the sequences against the human genome (Genome Reference Consortium Human Build 38, GRCH38) and subsequently annotates the alignments against different databases, assigning one count for each RNA species in the case of read overlapping as stated in their guideline. For piRNA, piRNABank, piRBase and piRNA Cluster databases were used, while gtRNAdb was used for tRNA and gencode annotation for snRNA and snoRNA (Li et al., 2020). Data generated in RNA-seq was processed in a similar manner. The quality of the sequences was analyzed with FASTQC, and the first 10 bases of the reads were removed with cutadapt v3.4, as well as those bases that did not match a minimum *Phred score* of 20 at the 3’ end. Alignment was performed with RSEM using HISAT2 as aligner (Li and Dewey, 2011). The filtered reads were aligned to the human transcriptome reference (GRCH38) to estimate the abundance of each gene in counts and its credibility interval. This value allows us to calculate the coefficient of interquartile variation of transcripts per million for each gene, filtering out genes with lower variation (coefficient of interquartile variation < 0.1).

### 2.5 Differential expression analysis and functional enrichment

The expression matrices generated in the annotation step were analyzed in the RStudio program (v1.4.1743–4 Evergreen Rose) based on R language (v 4.2.0). Separate count matrices were generated for each RNA species (miRNA, piRNA, tRNA, snRNA, snoRNA, mRNA). Transcripts of sncRNA that did not reach 5 counts in at least half of the participants were removed. The same filter with a threshold of 20 count was applied to the mRNA expression matrix. Expression data matrices were imported to the R environment with *tximport* (Soneson et al., 2015). Next, the R package *DESeq2* was employed to normalize the count matrices and calculate expression differences for each transcript through a negative binomial distribution (Love et al., 2014). The probability of differential expression is calculated with a Wald test, and *p*-values are adjusted with a Benjamini and Hochberg method. Elements were considered differentially expressed when adjusted *p-*values (*p-*adj) were lower than 0.05 and the transcripts presented an absolute fold-change (FC) greater than 2. Inter-sample distance matrices, principal component analysis (PCA), and complementary multivariate outlier detection metrics, including Hotelling’s T², Q residuals (squared prediction errors), and Mahalanobis distance, were used as exploratory approaches to identify potential outliers prior to differential expression analysis. Potential outliers were defined as samples exceeding the 95% threshold for both Hotelling’s T² and Q residuals and/or the χ²-derived critical value for Mahalanobis distance. Samples identified by at least two of these detection metrics were considered robust multivariate outliers.

To explore the biological effects of mRNA expression changes we performed a gene set enrichment analysis (GSEA) with the web-based GeneTrail 3 Transcriptomics workflow (Gerstner et al., 2020). Gene scores were calculated with p-values and FC obtained from the differential expression analysis using the following formula:


$$Gene\;score=\;-log_{10}(p-adj)\cdot log_{2}(FC)$$

All those biological terms were considered enriched when the False Discovery Rate (FDR) was below 0.05. Gene sets used in the GSEA were retrieved from different databases, including Gene Ontology (GO), Kyoto Enciclopedia of Gene and Genomes (KEGG) - Pathways, Reactome, Wikipathways, Transcription Factor Database (TRANSFAC), miRTarbase, Molecular Signatures Database (MSigDB), Protein Family Database (PFam) and Pharmacogenomics Database (PharmGKB).

### 2.6 miRNA-target network analysis

To study the relationship between miRNA and mRNA, expression data of both transcriptomic compartments were explored. First, differentially expressed miRNA and mRNA (FC = 1.5, *p*-adj < 0.05) between ECs and each of the non-controller groups (NT or ART) were selected. Both expression matrices were confronted and miRNA-mRNA pairs showing inverse expression changes were chosen and Spearman’s correlation coefficient (ρ) was calculated (R package *ggcorrplot2*). Subsequently, miRNA-target pairs were identified using the experimentally validated interaction database Tarbase, and miRTarBase (Karagkouni et al., 2018; Huang et al., 2022). We constructed a miRNA-mRNA interaction network Cytoscape v3.9.1 software. In addition, to identify the main biological pathways in which miRNA-mRNA pairs are involved we performed an over-representation analysis (ORA). We applied a hypergeometric test using GO biological processes, KEGG and Reactome as reference databases in the web tools KOBAS and TargetMine (Bu et al., 2021; Chen et al., 2022). Pathways are considered overrepresented with an FDR-adjusted p-value < 0.05, a minimum of 2 miRNA-regulated genes present in the pathway, and a representation ratio > 3%.

### 2.7 Models for *in vitro* functional assays

#### 2.7.1 Cells

CEM.NKR-CCR5 cell line (NIH AIDS Research and Reference Reagent Program, N° Cat: 4376), kindly provided by Dr. Sanchez-Palomino from IDIBAPS, was cultured in RPMI 1640 medium (Lonza™, BE12-115F, Biowhittaker™, Basel, Switzerland), supplemented with 10% decomplemented fetal bovine serum (Gibco™, 10270106, Thermo Fisher Scientific), and Penicilin/Streptomycin (100 UI/ml, 100μg/ml) (Lonza™, 09-757F) at 37°C and 5% CO_2_.

#### 2.7.2 Transfection and miRNA silencing

Exponentially growing cells were transfected with synthetic inhibitors for the human miRNA candidates involved in HIV restriction with the use of a Gene Pulser Electroporator (Bio-Rad, Hercules, CA, USA). Briefly, 6×10^6^ cells were collected in 500 µl RPMI 1640 medium and subsequently added 300 pmol of each miRNA inhibitors, either individually or in combination. The cells were transfected in a 4-mm electrode gap (BioRad) at 240 V, 1300 µF and maximum resistance. Upon transfection, cells were cultured in supplemented RPMI 1640 at 2×10^6^ cells/ml for 24h. The miRCURY LNA miRNA inhibitors (Qiagen Iberia SL) miR-99b-5p (410402599-ADC), miR-125a-5p (4104101665-ADB), and a scrambled control that do not target any known human miRNA (4100199006-ADB) were conjugated in the 5’-end to 6-carboxyfluorescein (FAM) to evaluate transfection efficiency 24h post-transfection by flow cytometry. Briefly, a portion of transfected cells were centrifuged at 350 *g* for 5 minutes and washed twice with phosphate-buffered saline. Dead cells were dyed with LIVE/DEAD™ fixable violet 405 nm (Thermo Fisher Scientific) following manufacturer’s instruction. Cells were washed twice with phosphate-buffered saline and fixed with 1% paraformaldehyde for 1h at 4°C. Then, fluorescence was measured in a Fortessa Flow Cytometer (BD Biosciences, San Jose, CA, USA). Flow cytometry data was analyzed using the FlowJo v9 software (BD Biosciences).

#### 2.7.3 Evaluation of mRNA and miRNA expression by RT-qPCR

Upon 24 h post transfection total RNA was isolated from transfected cells to evaluate miRNA and their target mRNA expression. One million cells were collected and washed with phosphate-buffered saline. Cell pellet was lysed with Qiazol (Qiagen Iberia SL), and RNA was isolated with miRNeasy mini kit columns (Qiagen Iberia SL) following the manufacturer’s instructions. Quantification of miRNAs was carried out as follows: 100 ng RNA was converted into cDNA with the miRCURY LNA RT (Qiagen Iberia SL) under the following conditions: 42°C for 60 min and 95°C for 5 min, according to the manufacturer’s instructions. The expression of miR-125a-5p, and miR-99b-5p miRCURY were measured with specific primers’ set included in the LNA PCR assay (Qiagen Iberia SL) according to the kits’ instructions. qPCR cycle conditions were 95°C for 2 min; 40 cycles: 95°C for 10 s, and 56°C for 60 s; and a final melting curve. The miRNAs -93-5p and 25-3p were used as endogenous control, upon selection with NormiRazor web tool and experimental validation. Candidate target genes were selected *a priori* from the miRNA–mRNA interaction network based on their involvement in pathways of interest and the existence of experimentally validated interactions in miRTarBase and TarBase. To maximize biological interpretability, target expression was only assessed in the inhibitor condition of the corresponding validated regulator miRNA. The level of expression of the miRNA-targets was assessed from 100 ng of total RNA, converted into cDNA with the GoScript™ Reverse Transcription System (Promega, Madison, WI, USA), with the random hexamer primers with a temperature program of 25°C for 5 min; 45°C for 60 min; 70°C for 15 min following the manufacturer’s instructions. Upon cDNA synthesis, qPCR reaction was performed with the SYBR^®^ Select Master Mix (Applied Biosystems, Foster City, CA, USA) and specific primers (**Supplementary Table 1**) at 95°C for 10 min; 40 cycles: 95°C for 15 s, and 60°C for 60 s; and a final melting curve. *ACTB* was used as the housekeeping gene. Reverse transcription reactions were performed in a C1000™ Thermal cycler (BioRad), and qPCRs were carried out in a StepOnePlus™ (Applied Biosystems). Results from qPCR were analyzed by Livak-Smittchgen method: the Ct was obtained by second derivative method and gene expression was normalized with the Ct values of endogenous controls (miRNA -93-5p, and 25-3p for miRNAs and *ACTB* for mRNAs) (Livak and Schmittgen, 2001). The 2^-ΔΔCt^ value was obtained using the miRNA scrambled control transfected cells as reference with the StepOne™ Real-Time PCR System.

#### 2.7.4 Infection assays

Infectious supernatants of NL4.3-Ren strains were obtained from a calcium phosphate transfection of 293T cells (ATCC^®^ CRL-3216™) with a pNL4.3-Ren plasmid, as previously described (Garcia-Perez et al., 2007). Subsequently, volumes corresponding to 100, 000 and 250, 000 RLU, produced by 10^5^ CEM cells, were used in triplicate to infect 1×10^5^ miRCURY-transfected CEM cells. The infected cells were cultured in supplemented RPMI 1640 at 37 °C with 5% CO_2_. At 48h post-infection cells were lysed and viral replication was assessed by measuring luciferase activity (Renilla Luciferase assay, Promega) using a 96-well plate luminometer LB 960 Centro XS^3^ (Berthold Technologies, Oak Ridge, TN, USA). Cell viability was measured upon infection using a CellTiter-Glo^®^ Luminescent Cell Viability Assay (Promega). Briefly, 10^5^ cells were incubated in duplicates in CellTiter-Glo^®^ reagent for 10 min at room temperature and the luminescent signal was registered in the luminometer LB 960 Centro XS^3^ (Berthold Technologies). The average RLU from viability assays was used to normalize each transfection and infection conditions.

### 2.8 Statistical analysis and representation of figures

For the description of the clinical variables of the participants, the median was used as a measure of central tendency and the interquartile ranges [Q1 lower quartile - Q3 upper quartile] were calculated to show the variability and dispersion in each of the groups. To calculate the differences of these variables between the different groups, the Kruskal-Wallis’ test is used for continuous variables and Fisher-exact test for discrete variables. Differences in mean gene expression level between miRNA inhibitor transfected cells were analyzed with a Mann-Whitney U test, and data was presented as mean ± SEM. Differences in infection assays were evaluated with a Wilcoxon test, geometric mean was calculated as the n^th^ root of the product of all experimental replicates and expressed with the 95% confidence interval. P-values were represented as * for *p* < 0.05, ** for *p* < 0.01, and *** for *p* < 0.001. Figure representation was performed with the R package *ggplot2.*

## 3 Results

### 3.1 Clinical characteristics of participants

The clinical characteristics of the participants are shown in **Table 1**. The cohort of study was mainly composed of men that acquired HIV infection through sexual intercourse, being most representative men who have sex with men. Noteworthy, the EC group had a slightly higher proportion of women than the rest of groups (*p*-value = 0.38, Fisher exact’s test). Viral load showed differences among groups as expected due to higher viral load in NT (*p*-value = 5.6×10^-6^, Kruskal-Wallis’ test). All groups had a median CD4+ T cell count close to 600 cells per mm^3^ of blood (*p*-value = 0.743, Kruskal-Wallis’ test) and similar CD4/CD8 ratio (*p*-value = 0.267, Kruskal-Wallis’ test). We did not find differences (*p*-value = 0.82, Fisher exact’s test) in the frequency of MHC alleles HLA-B57 and HLA-B27, which are associated with a better response to HIV infection. None of the participants presented active Hepatitis C Virus (HCV) coinfection at the time of sampling. Among the controller’s groups six EC, and five VC participants received ART between 1 and 9 years after sampling.

**Table 1:** Clinical characteristics from participants included in the study.

Group	EC	VC	ART	NT
Sample number	10	6	9	8
Sex (Male)	6 (60.0%)	6 (100.0%)	7 (77.8%)	7 (87.5%)
Transmission route				
MSM	4 (40.0%)	6 (100.0%)	4 (44.4%)	6 (75.0%)
HTSX	3 (30.0%)	0 (0.0%)	2 (22.2%)	1 (12.5%)
IDU	2 (20.0%)	0 (0.0%)	1 (11.1%)	0 (0.0%)
CBP	1 (10.0%)	0 (0.0%)	0 (0.0%)	0 (0.0%)
Unknown	0 (0.0%)	0 (0.0%)	2 (22.2%)	1 (12.5%)
Viral load (cp/ml)	37 [36-83]	1441 [1020 - 2101]	36 [36-37]	22586 [17485-34007]
CD4+ T cell count (cells/mm^3^)	706 [573-801]	610 [527-666]	594 [510-956]	647 [562-810]
CD8+ T cell count (cells/mm^3^)	946 [602-1314]	1188 [640-1208]	629 [548-767]	1132 [840-1331]
Ratio CD4/CD8	0.85 [0.52-1.16]	0.50 [0.43-0.95]	1.10 [0.80-1.40]	0.60 [0.50-0.67]
Nadir CD4+ (cells/mm^3^)	390 [347-454]	393 [301-479]	277 [269-383]	417 [368-470]
Years from HIV diagnosis	12.0 [8.1-14.0]	6.5 [3.8-8.8]	10.7 [5.7-16.9]	4.1 [2.3-6.6]
HLA -B57/-B27 alleles presence	3 (30.0%)	1 (16.6%)	3 (37.5%)	2 (22.2%)

Continuous variables are represented as median (IQR). Qualitative variables are represented as number (%). EC, Elite Controllers. VC, Viremic controllers. ART, Non-controllers under antiretroviral therapy. NT, Non-treated (or Naïve) non-controllers. cp/ml, HIV RNA copies/ml of blood. MSM, Men who has sex with men intercourse. HTSX, Heterosexual intercourse. IDU, Intravenous drug users. CBP, Contaminated blood products. Presence of HLA-B protective alleles is considered positive when one of the mentioned alleles is at least present in heterozygosis (**Supplementary Table 2**).

### 3.2 Elite controllers have a characteristic sncRNA expression profile

To inspect elite controllers sncRNA expression profile we performed RNA-Seq on sncRNA from isolated CD4+ T cells from PWH including natural HIV controllers (EC and VC) and non-controllers (NT and ART). Next, we evaluated the expression of miRNA, piRNA, tRNA, snRNA, and snoRNA, following a differential expression analysis among the study groups. The sncRNA libraries yielded an average (± standard deviation) of 5, 794, 508 ± 1, 198, 825 reads per participant. After the filtering step, an average of 70.7% of the reads was retained, leaving out reads mainly identified as adapters or long non-coding RNA. Regarding the annotation of the filtered sequences, around 18% annotate with miRNA (790, 210 ± 327, 695 reads/participant), 40% for tRNA (1, 737, 677 ± 538, 573 reads/participant), 6% annotate for piRNA (284, 162 ± 133, 389 reads/participant), 1% for snRNA (39, 405 ± 16, 966 reads/participant) and 5% for snoRNA (220, 883 ± 81, 134 reads/participant). About 30% of the sequences subjected to alignment failed to annotate any sncRNA elements. An average of 358 ± 42 miRNA, 201 ± 11 tRNA, 459 ± 58 piRNA, 241 ± 48 snRNA, and 273 ± 7 snoRNA were identified per participant with at least 5 uniquely aligned reads.

In the exploratory analysis, participant NT-7 was identified as a multivariate outlier. Across multiple independent sncRNA datasets, NT-7 showed consistent separation in PCA plots and repeatedly exceeded Hotelling’s T² and Mahalanobis distance thresholds. The participant introduced a substantial influence on the covariance structure of the datasets and on downstream differential expression analyses, indicating a leverage effect markedly greater than that observed for any other individual. To avoid bias introduced by a single highly influential observation, NT-7 was excluded from all sncRNA analyses. Participant ART-3 displayed a similar behavior within the piRNA and tRNA datasets and therefore was excluded exclusively from those analyses (**Supplementary Figure 1**). Correspondingly, differential expression analysis was performed before and after multivariate outlier removal, obtaining consistent results in the direction and the magnitude of sncRNA expression changes (**Supplementary Tables 3**; **4**). We found no differential in any sncRNAs associated with treatment administration except for 8 tRNA (-Glu-TTC) overexpressed in ART compared to NT (**Supplementary Table 3**).

Focusing on the tRNAs expression profile in elite controllers, we found two glutamic acid-associated tRNAs (tRNA-Glu-TTC) repressed in ECs compared to ART. These tRNAs correspond with those found previously overexpressed in ARTs versus NTs (**Figure 1**). Notably, the tRNA-Lys-CTT-3–1 is overexpressed in ECs versus ART L2FC (Log_2_[FC]) = 3.01, FDR<0.01), and ECs versus NTs (L2FC = 4.32, FDR = 0.01). It is also observed that there is a trend towards overexpression of tRNAs associated with lysine, glycine, threonine or arginine (CCG or TCG anticodons), whereas tRNAs associated with alanine, selenocysteine, proline or arginine (ACG anticodons) are repressed in ECs compared to NT (**Figure 1**).

**Figure 1:**
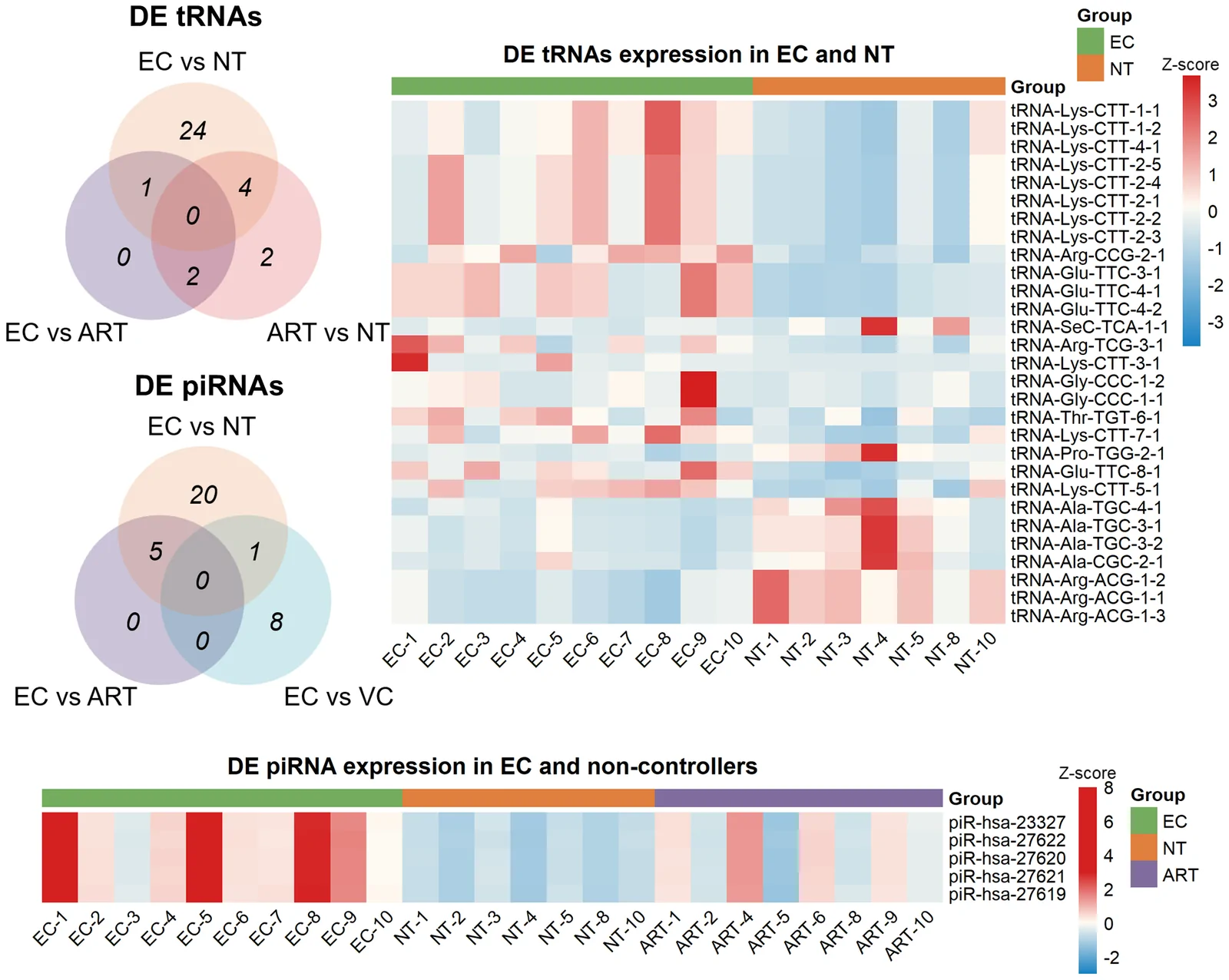
Elite controllers present differentially expressed tRNA and piRNA. Venn diagrams show DE tRNA and piRNA in common among comparisons. Each circle represents a comparison. Heatmaps of selected intersections for tRNA (EC vs NT), up, and piRNA (EC vs NT + EC vs ART), down, are shown. Participants EC-1 and EC-5 present extremely high values of piRNA expression, and are represented as the top shade of red.

Characterizing the expression profile of piRNAs we found 5 and 26 DE piRNAs in EC when compared to ART, and NT groups, respectively (**Figure 1**). The 5 piRNAs found overexpressed in EC vs ART (piR-hsa-23227, -27622, -27621, 27620, -27619, average L2FC = 2.15) are also expressed to a higher extent in EC compared to NT (average L2FC = 1.27) (**Figure 1**). Additionally, there are 8 overexpressed piRNAs between ECs and VCs (**Supplementary Table 3**). and the repression of piR-hsa-2138 in ECs (L2FC=-0.99, FDR = 0.03) which also is repressed in EC vs NT (L2FC=-0.86, FDR = 0.03).

For snRNAs and snoRNAs DE elements were only found in ECs compared to NTs. The snRNAs RNA6ATAC40P (L2FC = 1.38, FDR = 0.01), RNU6ATAC31P (L2FC = 1.38, FDR = 0.01), and RN7SK (L2FC = 0.77, FDR = 0.01) are overexpressed, while RNU5D-1 (L2FC=-0.67, FDR = 0.05) and RNU5F-1 (L2FC=-0.88, FDR = 0.02) are repressed in ECs. The only detected DE of snoRNAs is the overexpression of SNORA6 (L2FC = 1.07, FDR = 0.01) in ECs versus NT. Results from all sncRNAs differential expression analysis can be accessed at **Supplementary Table 3**.

### 3.3 Elite controllers display a specific miRNA expression profile

We evaluated the presence of a miRNA expression signature in HIV control comparing EC and VC against non-controllers’ group (**Supplementary Table 4**). Similarly to the rest of sncRNA, we explored differences between NT and ART PWH, and no DE miRNA was found, suggesting that detectable viremia does not alter the miRNA expression profile of the majority of circulating CD4+ T cells. Three miRNAs: miR-125b-2-5p, miR-125a-5p, and miR-99b-5p were differentially expressed (**Figure 2**) between EC and ART groups. The latter two of these miRNAs also have lower expressions in ECs when compared to NT group, as well as miR-221-5p, -501-3p, 1271-5p, -1246, and -151b. Comparison of VCs with EC or ART has not shown DE miRNA, while the comparison between VC and NT showed four downregulated miRNAs in VC. Three of these miRNAs (miR-99b-5p, miR-221-5p, miR-501-3p) are also less expressed in ECs in other comparisons supporting the potential protective role of these miRNAs on the control of HIV replication.

**Figure 2:**
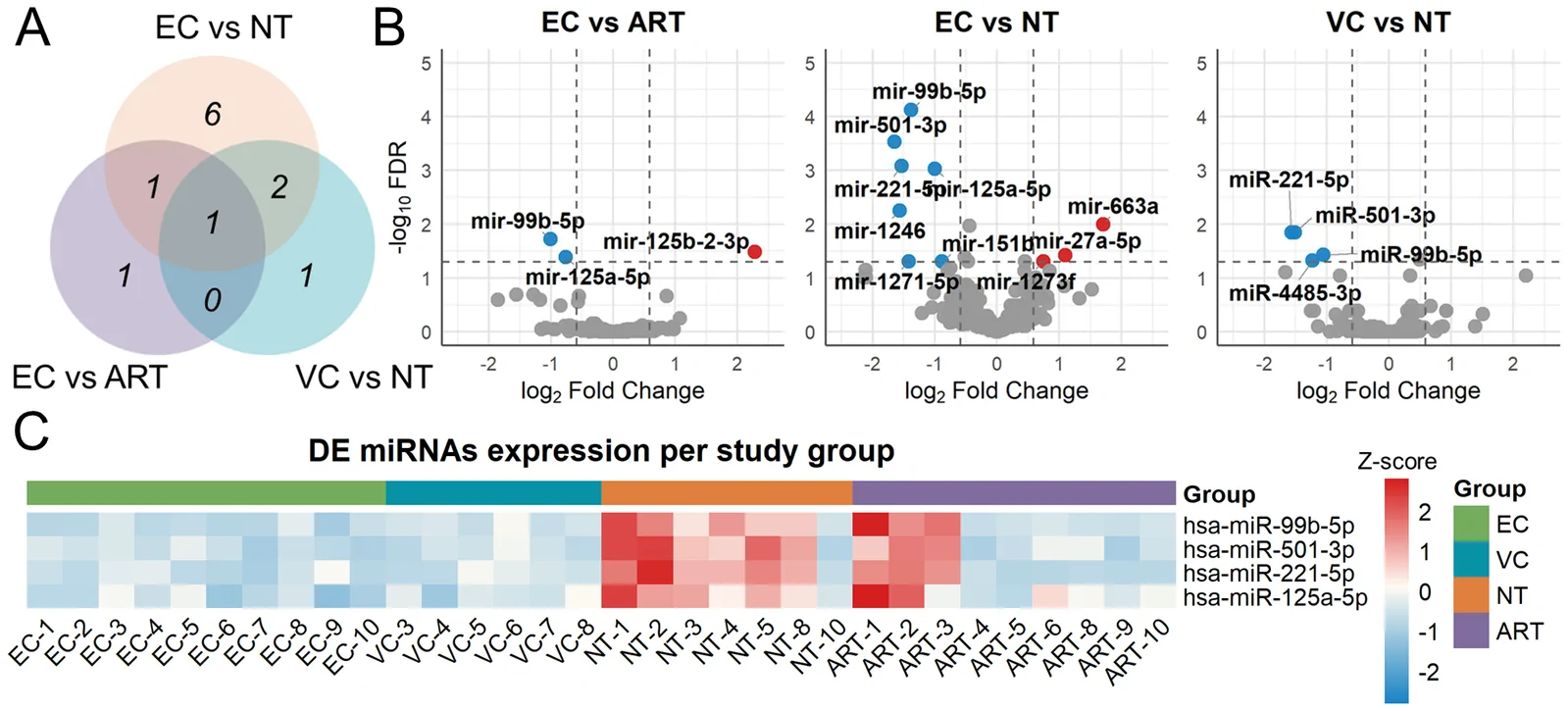
Elite controllers present common downregulated miRNAs when compared to non-controllers. **(A)** Venn diagrams of DE miRNA in each comparison. **(B)** Volcano plots of each comparison. miRNAs are represented by a point. Blue: downregulated; red: upregulated. **(C)** Heatmap of selected miRNA present in the intersected area of Venn diagram. The color bar represents the Z-score.

### 3.4 Transcriptomic signatures involve immune activation, inflammation and metabolic alterations in elite controllers

To better address the transcriptomic landscape of CD4+ T cells, we also performed an RNA-Seq in the same RNA extraction. Briefly, the RNA-Seq data rendered on average (± SD), 43, 351, 531 ± 19, 450, 903 reads per participant and once trimmed and aligned to the human transcriptome, a total of 9, 968 genes were identified with an annotation credibility interval above 90%.

To characterize the transcriptional state of CD4+ T cells in ECs at the functional level, a GSEA was performed. In the EC vs ART comparison, a total of 243 enriched terms were obtained with an FDR-adjusted probability (FDR)<0.05 while comparing EC with NT 414 terms were enriched (**Supplementary Figure 2**). To find the common terms and transcriptomic changes occurring in EC versus any of the non-controller groups, we represented the intersection of both enrichment analyses (**Figure 3**). A total of 88 terms were obtained in both analyses, observing an increment in the expression of genes involved in the signaling by the TNF-α pathway via NF-κB, p53 signaling pathway, and inflammatory response (**Supplementary Table 5**). Some of the terms enriched in genes reducing their expression are the general transcription pathway, or genes containing KRAB-box motifs and Zn-fingers associated C2H2-motifs. Of note, also there is a greater expression of genes controlled by the transcription factors SP1, the JUN family, SP3, CREB, 1 or CEBPB. In addition, there is a general increase in ECs in terms related to cell activation, apoptosis, cell death, response to hypoxia and UV radiation, and elements of oxidative stress response such as the NRF2 pathway or the selenium micronutrient network. Regarding metabolic pathways, genes involved in carbohydrate metabolism, and cholesterol homeostasis terms are also upregulated.

**Figure 3:**
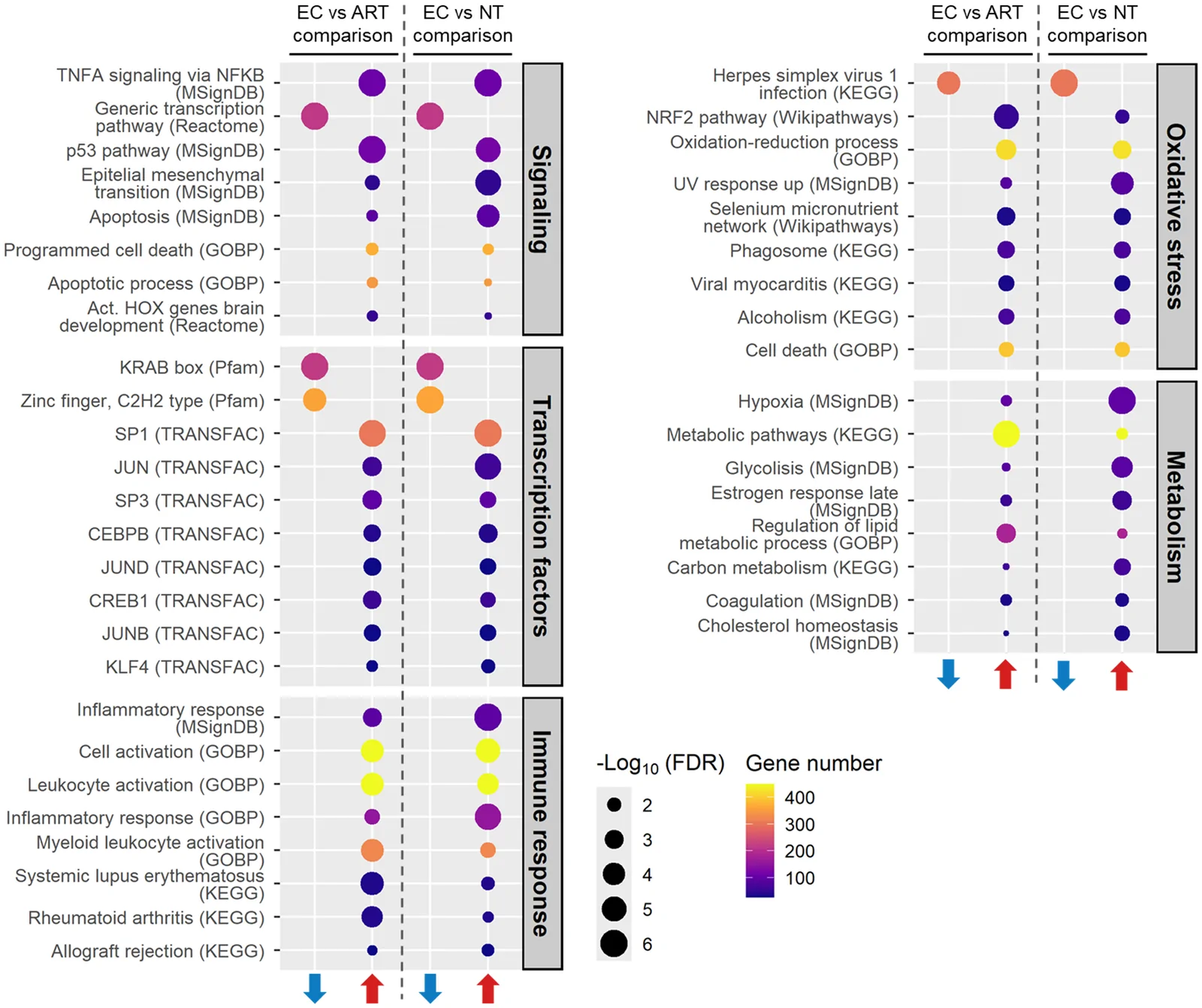
Altered biological processes in the transcriptomic signature of elite controllers compared to non-controllers. The GSEA plot represents those terms enriched in genes scored by their fold-change and statistical significance for both comparisons, EC vs ART and EC vs NT. Blue downward arrows indicate that those terms are enriched in repressed genes in EC while red upward arrows indicate terms enriched in upregulated genes in EC. Each term’s database of origin is indicated between brackets. Most relevant terms regarding functionality are shown in the picture.

Furthermore, we performed a differential expression analysis and found that there were 106 DE genes (DEG) between EC and ART groups and 104 DEG in the EC and NT comparison (**Supplementary Table 6**). Of those, 26 DEG were found to be commonly deregulated (**Supplementary Figure 3**). The overrepresentation analysis (ORA) using the DEG found in each comparison reproduces the previous results obtained by GSEA in both cases, showing that DEG-enriched terms are related to immune response and inflammation, p53 signaling, and the metabolism of sterols (**Supplementary Figures 4**, **5**). Of note, the differential expression analysis did not find any DEGs between VCs and any other of the study groups.

### 3.5 EC miRNA expression is correlated to the expression of key genes involved in those processes

To better understand the transcriptomic alterations that could be led by miRNA expression changes, we selected the DE miRNA and DEG from each comparison and built a miRNA-mRNA interaction net. We inspected the experimentally validated miRNA-mRNA pairs available in public databases that presented a correlation based on their expression data and found that in EC vs ART network there were 41 DEG influenced by the expression of miR-99b-5p and miR-125a-5p (**Figure 4A**). Several of these genes had already been identified in the intersection of DEGs between EC vs ART and EC vs NT, such as *FLNA, DPM2, CDKN1A, BBC3, DUSP1*, and *SOD2*. To visualize the processes regulated by this set of genes, we performed an ORA (**Figure 4B**, **Supplementary Table 7**). Correspondingly with the results we obtained in the RNA-Seq ORA, several of the enriched terms were related to p53 signaling pathway, endogenous sterols, activation of matrix metalloproteinases, and metabolic disorders of biological oxidation enzymes (**Figure 4C**), suggesting that the major observed changes in the mRNA analysis could be regulated by the expression of miR-99b-5p and miR-125a-5p in EC. In the network generated by EC vs NT comparison data, there were 86 DEGs regulated by 7 of the DE miRNAs, also involving miR-99b-5p and miR-125a-5p (**Figure 5A**). Again, the results display common enriched terms with the previous ORA, related to response to infection and regulation of metabolism (**Figure 5B**, **Supplementary Table 7**). In addition, there are several overrepresented pathways of interest, such as Fc gamma-receptors dependent phagocytosis, TP53 regulates transcription of cell cycle genes, OAS antiviral response, or BH3-only proteins associate with and inactivate anti-apoptotic BCL-2 members, which highlight an alteration in cell progression and apoptosis and in the antiviral response in CD4+ T cells of ECs compared to NTs (**Figure 5C**). In this manner, we observe that a large proportion of cellular processes altered in EC are also related to the modification of the expression of the DE miRNAs we found associated with EC.

**Figure 4:**
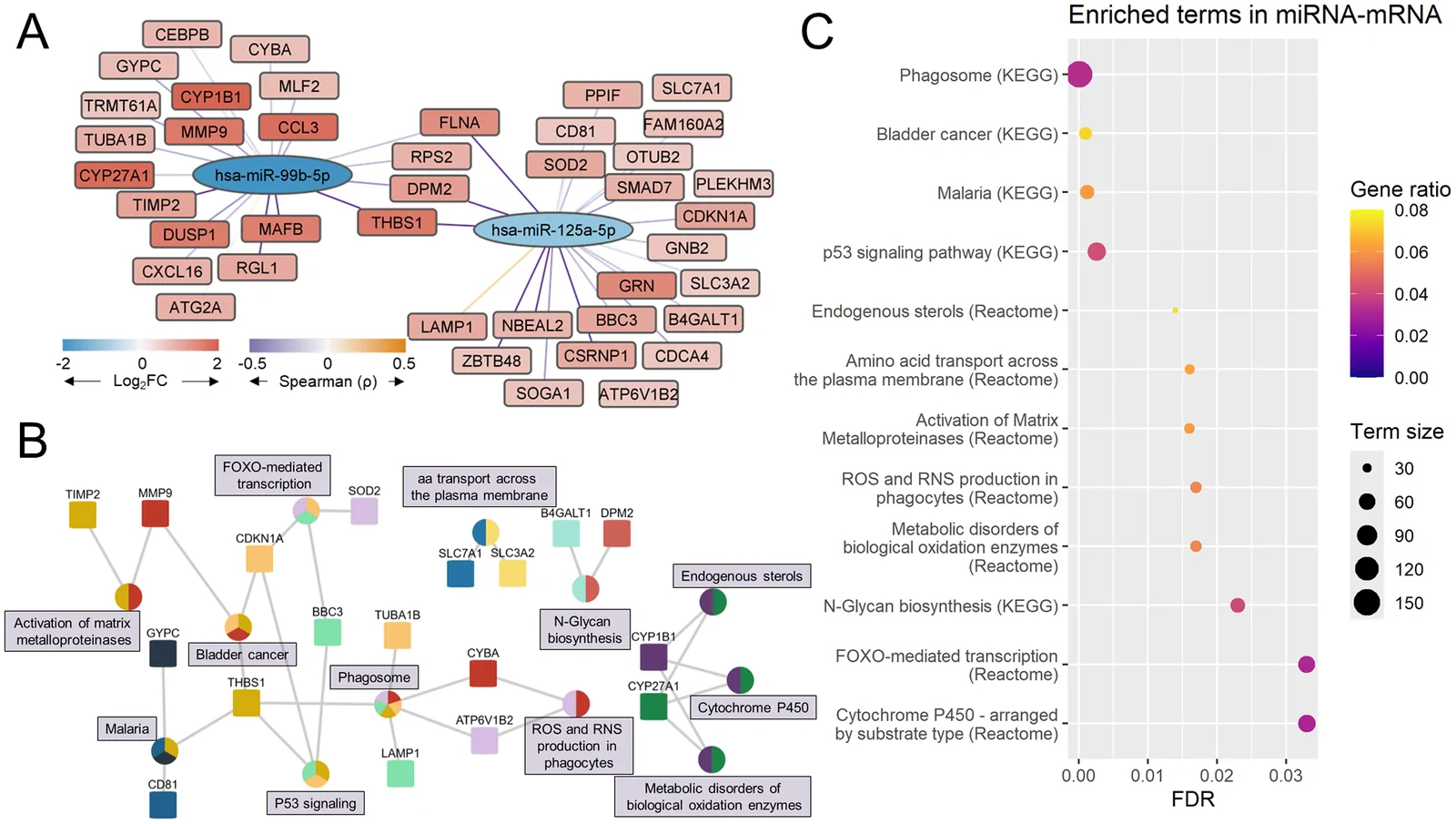
EC vs ART DE miRNAs correlate with DEG implicated in disease and immune response. **(A)** miRNA-mRNA interaction network. The nodes represent miRNAs (circles) and genes (squares) that are differentially expressed (DE) (|LFC| > 1, FDR < 0.05). The color scale of the genes represents the L2FC value, while the colors of the segments represent the Spearman coefficient value. Interactions were retrieved from databases of experimentally validated miRNA-mRNA pairs. **(B)** Network displays the enriched terms and genes that belong to each term. The terms are represented as colored circles according to the genes that compose them (squares). **(C)** Overrepresentation analysis of genes found in the miRNA-mRNA interaction network. Dots’ size refers to the number of genes that compose each functional term, while the color of the dot indicates the percentage of DEG referred to term size. Figure shows only Reactome and KEGG terms for visualization purposes while GO Biological Processes can be accessed in **Supplementary Table 7**.

**Figure 5:**
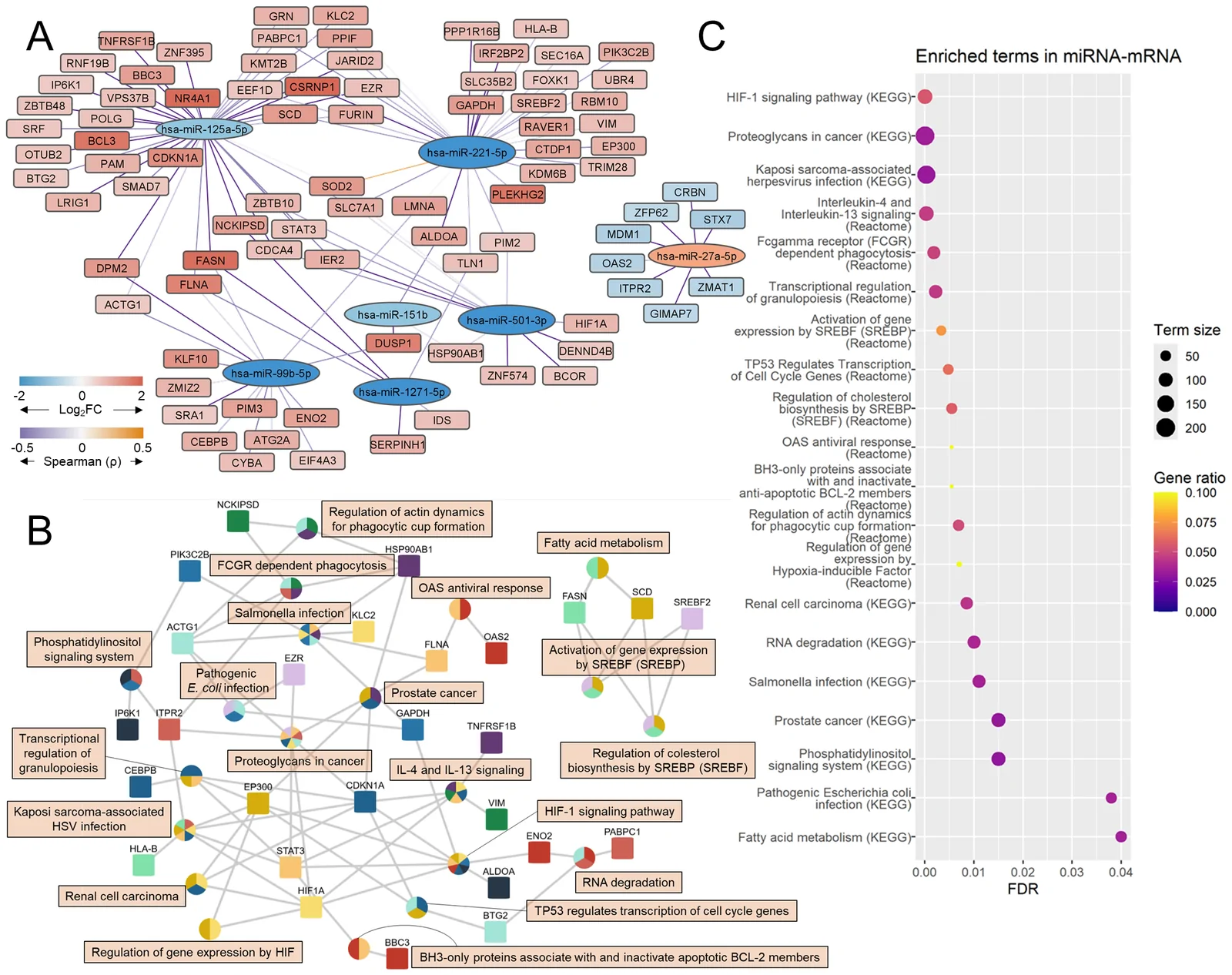
EC vs NT DE miRNAs correlate with DEG implicated in immune response and lipid metabolism **(A)** miRNA-mRNA interaction network. The nodes represent miRNAs (circles) and genes (squares) that are differentially expressed (DE) (|LFC| > 1, FDR < 0.05). The color scale of the genes represents the L2FC value, while the colors of the segments represent the Spearman coefficient value. Interactions were retrieved from databases of experimentally validated miRNA-mRNA pairs. **(B)** Network displays the enriched terms and genes that belong to each term. The terms are represented as colored circles according to the genes that compose them (squares). **(C)** Overrepresentation analysis of genes found in the miRNA-mRNA interaction network. Dots’ size refers to the number of genes that compose each functional term, while the color of the dot indicates the percentage of DEG referred to term size. Figure shows only Reactome and KEGG terms for visualization purposes while GO Biological Processes can be accessed in **Supplementary Table 7**.

### 3.6 Modulation of miRNA associated with elite control reduces HIV infection

Overall, the results above suggest that miRNAs could be involved in the regulation of cellular processes in CD4+ T cells that are vital to the virus replication cycle. To explore the biological function of miRNAs associated with elite control in CD4+ T cells, we knocked down the expression of miR-99b-5p and miR-125a-5p in the CEM cell line by transfecting miRNA inhibitors, solely or in combination. Transfection efficiency was measured by flow cytometry obtaining 2.57% (1.21-3.93) (average – 95% confidence interval) for miR-99b-5p, 5.06% (1.75%-8.37%) for miR-125a-5p and 5.97% (4.13%-7.8%) for the combination of both. After transfection, the change in expression of the previously selected miRNAs and target mRNAs was measured. The inhibition of miRNA expression 24 hours after transfection reduced more than 90% of their expression using both inhibitors alone, and in combination (**Figure 6A**). In relation to the targets of these miRNAs, their expression tended to increase, with a fold change between 1.1 and 1.2 compared to the control (**Figure 6B**). However, the augmentation in target expression at the mRNA level is not as marked as the downregulation of their repressors upon inhibitors’ transfection. Next, we evaluated whether HIV-1 infection and replication were hindered in our CD4+ T cell model. Cells were infected at two doses with NL4.3-Ren 24h post transfection. Following 48 hours of infection viral production was reduced at both inoculation doses (**Figure 6C**). At the lowest dose, the reduction was 18.2% (3.6%-30.5%) (geometric mean [confidence interval]) for miR-99b-5p inhibitor, 18.4% (6.4%-28.9%) for miR-125a-5p inhibitor, and 22.0% (11.7-31.1%) for cells with both inhibitors combined. For the higher dose, reductions of 17.0% (5.5%-27.0%), 14.1% (5.5%-30.1%; p>0.05), and 20.7% (15.5-26.9%) were obtained for miR-99b-5p, miR-125a-5p, and miR-99b-5p plus miR-125a-5p inhibitors, respectively. Therefore, our data highlights that the repression of miR-99b-5p and miR-125a-5p has a positive effect on reducing viral replication in CEM cells.

**Figure 6:**
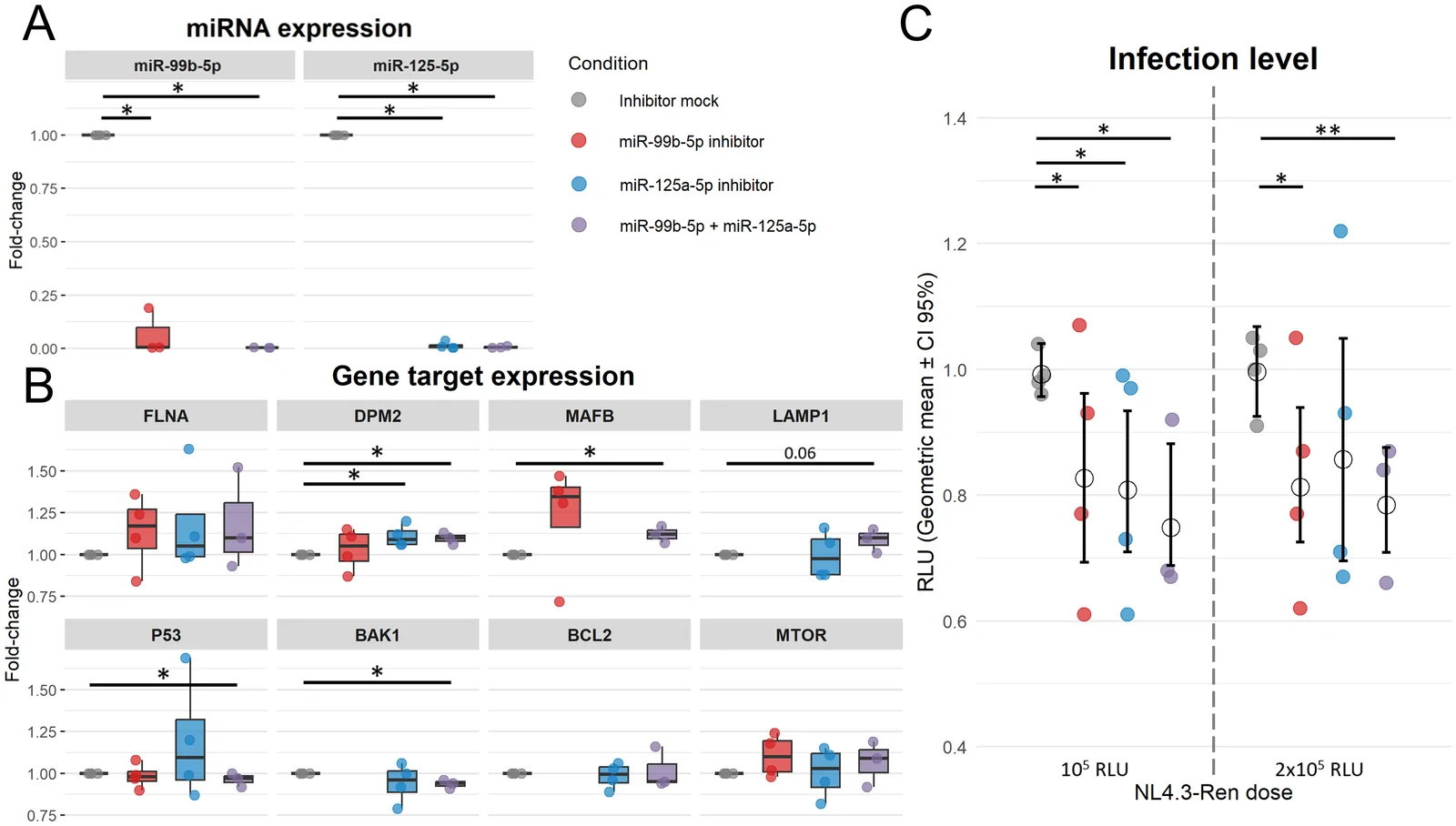
miR-99b-5p and miR-125a-5p downregulation reduce HIV infection. **(A, B)** miRNA and their gene targets expression 24h after inhibitor transfection, respectively. Boxplots represent the interquartile range (IQR) and whiskers extend to minimum and maximum values within 1.5xIQR. Fold-change is calculated by the 2^-ΔΔCT^ method. The gene targets were selected based on the results from the regulatory network representing pathways of interest, along with other relevant targets from enrichment results such as P53 (P53 signaling), MTOR (metabolic state), and BAK1, and BCL2 (apoptotic balance, only targets of miR-125a-5p). **(C)** Infection production upon miRNA inhibitor treatment. Blank points show the geometric mean while the bars indicate the 95% confidence interval (CI). Relative light units are normalized by viability. Points represent independent biological replicates (n = 3–4 per condition), each corresponding to the mean of three technical replicates. Statistical analysis was performed with a Wilcoxon matched-pairs signed rank test for paired groups and Mann-Whitney test for unpaired groups. *p ≤ 0.05, **p ≤ 0.01.

## 4 Discussion

This study provides the first characterization of a set of small non-coding RNA expression profile in CD4+ T cells from HIV controllers. We identified distinct signatures of tRNAs and piRNAs in EC compared to non-controllers PWH. tRNAs and tRNA-derived fragments (tRFs) possess non-canonical functions beyond their known role in translation. These include transcriptional and translational regulation, post-translational modification, and response to stress and pathogenic conditions (Keam and Hutvagner, 2015; Nunes et al., 2020; Su et al., 2020). HIV infection leads to phosphorylation of tRNA synthetases, such as lysyl-tRNA synthetase and glutamyl-prolyl-tRNA synthetase (EPRS1), disrupting multi-synthetase complexes and modulating cytoplasmic tRNA pools. EPRS1 release produces an antiviral effect (Lee et al., 2016), potentially explaining the differential tRNA-Glu expression seen among EC, ART, and NT groups. HIV also co-opts lysyl-tRNA synthetase to facilitate reverse transcription (Duchon et al., 2017). Remarkably, we found that tRNA-Lys-3 is 6-8-fold overexpressed in EC compared with non-controllers. This is significant as double-stranded RNAs are originated from the HIV primer binding site for viral transcription and an 18 nt tRF derived from the 3’ end of tRNA-Lys-3. This dsRNA may be loaded into AGO2 and form a silencing complex that targets HIV mRNA (Yeung et al., 2009). Thus, the viral suppression in ECs might be partly due to an RNA interference mechanism, guided by the 18 nt tRF derived from the overexpressed tRNA-Lys-3 in EC. Other tRNA-Lys isoacceptors (-1, -2, -4, and -5) were also upregulated in ECs compared to NTs and contributed to group classification in sPLS-DA analysis. While tRNA-Lys-1, 2 facilitate nuclear import of the preintegration complex (Zaitseva et al., 2006), their distinct 3’ sequences prevent them from acting as primers in reverse transcription (Kang et al., 1999). Altogether, these findings underscore the multifaceted role of tRNAs in HIV replication.

This work also reports a unique piRNA signature in elite controllers. We identified five differentially expressed piRNAs, with nearly identical sequences, common to both EC comparisons. These originate from six genomic loci and are associated with tRNA-Lys-CTT repeats, consistent with the tRNA-Lys overexpression previously discussed. Although piRNAs are described to regulate gene expression via epigenetic modifications, mRNA silencing, and post-translational editing (Jia et al., 2022), no targets have been experimentally validated for RISC-mediated piRNAs silencing and their specific mechanism of action in HIV infection remains to be elucidated. Few differences were observed in snRNA and snoRNA expression. Most strikingly, we observed the upregulation of RN7SK in ECs. This snRNA inhibits HIV transcription by inactivating CDK9 and preventing p-TEFb recruitment to the viral promoter (Yang et al., 2001; Egloff et al., 2006). This finding is consistent with previous reports of CDK9 inhibition in ECs via p21 overexpression, reinforcing its critical role in suppressing viral replication (Chen et al., 2011). Among snoRNAs, only SNORA6 was upregulated in ECs vs NTs. As an H/ACA box snoRNA, it guides pseudouridylation of rRNA during ribosome biogenesis, especially in the nucleoli region (Deogharia and Majumder, 2018). These facts suggest a potential role in modulating the viral life cycle through ribosomal or RNA modifications. Collectively, these findings expand the landscape of non-coding RNAs associated with HIV elite control and open new avenues for investigating tRNA-, piRNA-, snRNA-, and snoRNA-mediated mechanisms of viral restriction.

The miRNA transcriptome comprises the last sncRNA compartment we have evaluated in this study. We examined the miRNA expression profile in the CD4+ T cells of elite controllers and its relationship with their gene targets’ expression. Differential expression analysis showed that CD4+ T cells from ECs had 3 DE miRNAs compared to the ART group, while up to 10 DE miRNAs were obtained in comparison with the NTs. Observing the similarities between both comparisons, ECs showed a common pattern of decreased expression of miR-99b-5p and miR-125a-5p compared to the ART and NT groups individually. It should be noted that in the VC vs EC comparison, no differences were found in miRNA expression, but in the VC vs NT comparison, a decrease in the expression of miR-99b-5p, miR-221-5p, and miR-501-3p was observed. There are previous articles that report changes in the expression of these miRNAs in HIV infection, such as the repression of miR-125a-5p in PBMC from EC compared to VC and PWH under ART (Egaña-Gorroño et al., 2014), or the overexpression of miR-221-5p in macrophages upon HIV infection *in vitro* (Lodge et al., 2017). Moreover, miR-501-3p was downregulated in plasma from young adults that acquired HIV infection vertically treated in their first year of life compared to a similar group that received treatment two years after being born (Tarancon-Diez et al., 2022). Altogether, these findings indicate that those miRNAs exert a role in the natural control of viremia.

To better assess the regulatory effect of these miRNAs on gene expression in the context of elite control, we concurrently studied the transcriptome of CD4+ T cells. We first addressed the transcriptome to evaluate changes in the expression profile of HIV controllers, obtaining a broader view through a GSEA employing the whole transcriptome and highlighting the genes of greatest interest in specific pathways via differential expression and ORA. Both GSEA and ORA yielded very similar enriched terms and pathways. Our analysis revealed an overrepresentation of p53 signaling in ECs. Following HIV infection, class I IFN activates p53, which elicits antiviral effects by inhibiting the LTR promoter, inactivating Tat, and inducing apoptosis—a term also overrepresented in our EC cohort (Duan et al., 1994; Perfettini et al., 2005; Cummins and Badley, 2010; Cooper et al., 2013; Yoon et al., 2015; Lee and Crotty, 2021). Key genes driving this pathway include the pro-apoptotic factor *BBC3*, *THBS1* (which binds gp120 to block CD4 engagement), and the established restriction factor *CDKN1A*/p21 (Crombie, 2000; Zhang et al., 2007; Chen et al., 2011; Leng et al., 2014; Shi et al., 2018; Chandrasekar et al., 2020). Similarly, terms related to inflammation and leukocyte activation were enriched in EC. This is supported by elevated NF-κB-mediated TNFα signaling and cytokine activity. While some studies report heightened inflammation in ECs compared to other groups (Hunt et al., 2008; Hatano et al., 2013; Li et al., 2015; Sperk et al., 2021), others report comparable levels (Bello et al., 2009; Bansal et al., 2015; Côrtes et al., 2018) underscoring the heterogeneous nature of ECs. Indeed, previous studies report that ECs show higher immune activation and inflammation levels (Pereyra et al., 2012; De La Torre-Tarazona et al., 2022), which has been linked to a greater prevalence of cardiovascular events in this group (Crowell et al., 2015). Systemic inflammation may be detrimental in the development of non-AIDS events; however, it could be favorable in local responses, activating immune mechanisms that assist in HIV control. We also observed the activation of HIF-1α signaling, a finding consistent with our data and previous reports in other EC cohorts (Svensson et al., 2021). Interestingly, HIF-1α stabilization under normoxic conditions has been shown to suppress provirus reactivation and LTR activity, suggesting a potential mechanism for viral control (Zhuang et al., 2020). Moreover, we detected an enhancement in pathways that mitigate oxidative stress, including NRF2 signaling, the selenium micronutrient network, and the response to UV radiation, which is supported by previously published data (Sperk et al., 2021; Svensson et al., 2021). This robust antioxidant response may be crucial for maintaining CD4+ T cell functionality under the heightened activation and persistent inflammation observed in EC. Finally, the transcriptomic data revealed reduced expression of KRAB and C2H2 zinc finger proteins in ECs. This finding is congruent with recent studies linking HIV control to transposable element regulation by KRAB zinc finger proteins (Singh et al., 2025). Notably genes encoding these proteins have been reported to preferentially harbour intact proviruses in the reduced reservoir of elite controllers and long-term ART-treated individuals (Einkauf et al., 2019; Lian et al., 2021).

To inspect whether the EC transcriptome is influenced by their characteristic miRNA profile, we integrated miRNA and mRNA expression data, focusing on experimentally validated targets that were also differentially expressed genes. In the EC vs ART comparison, decreased expression of miR-99b-5p and miR-125a-5p correlated with increased expression of 41 of their target DEGs. These genes are involved in processes key to viral control, including p53 signaling, reactive oxygen species (ROS) production, and phagosome—a pathway related to autophagy previously linked to HIV restriction via degradation of viral proteins like Tat (Nardacci et al., 2014; Sagnier et al., 2015). Similarly, in the EC vs NT comparison, a set of seven miRNAs (including miR-99b-5p and miR-125a-5p) targeted genes within the HIF-1α signaling and p53-mediated apoptosis pathways. This suggests miRNA regulation may support the metabolic activation and heightened sensitivity to cell death observed in ECs. In summary, the DE miRNAs in ECs are correlated with the changes in the expression of their gene targets, suggesting a regulatory role in pathways that are favorable for restraining HIV replication.

Finally, the results from our functional *in vitro* analysis further support the role of miR-99b-5p and miR-125a-5p downregulation hampering HIV replication. These miRNAs were selected as candidates because they were repressed in ECs versus both control groups. While we observed a moderate regulatory effect on some targets (i.e., *FLNA*, *LAMP1*, p53, *MTOR*), only *DPM2* (a regulatory subunit of the dolichol phosphate mannosyltransferase), and *MAFB* (a transcription factor with dual role in IFN-β signaling (Kim and Seed, 2010)) showed a statistically significant increase. The overexpression of these two targets does not appear to explain the inhibition of viral replication or fully recapitulate the signaling pathways altered in EC. Attempts to measure other relevant targets (*BBC3, CDKN1A, THBS1*) were unsuccessful, likely due to limitations of the *in vitro* model. As an immortalized T-cell line, CEM.NKR-CCR5 cells may not fully reproduce the regulation of genes involved in apoptosis and cell-cycle control observed in primary CD4+ T cells, potentially limiting the assessment of some miRNA-target interactions. Noteworthy, the ablation of our miRNA candidates appears to affect HIV replication independently of the infection dose.

The findings of this study should be interpreted in the context of a few limitations. The sample size is inherently small given the exceptionality of the EC and VC phenotype and, availability of samples of all groups. This limitation could explain, at least in part, the lack of significant differences between VC and non-controller groups. Our sncRNA alignment pipeline simultaneously aligns the reads with piRNA, tRNA, snRNA and snoRNA databases. If one read aligns with more than one sncRNA class, each small RNA will be assigned one count. Thus, piRNA signals sharing extensive sequence similarity with tRNA-derived fragments, particularly those associated with tRNA-Lys loci, should be interpreted with caution. Additionally, while our *in vitro* CD4+ T cell model supports a viral restriction phenotype, it may not fully replicate the *in vivo* environment of ECs. Therefore, the antiviral effects and regulatory interactions observed following miRNA inhibition should be interpreted within the context of this experimental system. Future studies in primary CD4+ T cells or *in vivo* models will be valuable to further validate the biological relevance of our findings. On the other hand, this study provides significant insights that advance our understanding of small non-coding RNAs underlying elite control and how they affect the transcriptional state of CD4+ T cells. Furthermore, our driven-result functional proxy highlights the antiviral role of two miRNAs despite the mechanism is still to be elucidated.

In summary, our work demonstrates that CD4+ T cells from elite controllers exhibit a distinct non-coding RNA signature, encompassing differential expression of piRNAs, tRNAs, snRNAs, and snoRNAs, which potentially could contribute to the inhibition of the HIV replication cycle. Furthermore, we identified a specific miRNA profile of elite control, defined by the significant downregulation of miR-99b-5p and miR-125a-5p. The repression of these miRNAs is inversely correlated with the overexpression of their validated gene targets, which may lead to modulating key immunological processes crucial to the EC phenotype. Compellingly, our functional data suggests that targeted repression of miR-99b-5p and miR-125a-5p supports a possible role in the restriction of HIV infection *in vitro.* As a whole, these data not only shed some light onto a novel layer of post-transcriptional actors participating in spontaneous HIV control but also pinpoint specific miRNA candidates for multitargeted interventions aimed at achieving a functional cure.

## Data Availability

The datasets presented in this study can be found in online repositories. The names of the repository/repositories and accession number(s) can be found below: https://www.ncbi.nlm.nih.gov/geo/, GSE313420.
