# Supplementary material for: sncRNA signatures underlying HIV-1 restriction in elite controllers
Source: Front Cell Infect Microbiol. 2026 Oct 20;16:1926123. doi: 10.3389/fcimb.2026.1926123 (PMC13727583; doi:10.3389/fcimb.2026.1926123)
Supplement: Supplementary file 1 [file SupplementaryFile1.zip › Supplementary Figure 1.docx]

**Supplementary Figure 1.Exploratory analysis and outlier identification across sncRNA datasets.** From left to right, panels show: 1) principal component analysis (PCA) including all samples, 2) PCA upon removal of multivariate outliers, 3) outlier detection metrics (X-axis: Hotelling T2; Y-axis: Q-residuals) with 95% significance threshold indicated by red lines, and 4) intersample distance matrices. Rows correspond to the different sncRNA datasets analyzed. Samples were defined as multivariate outliers when they fulfilled at least 2 of the following criteria: a) exceeding the 95% threshold for Hotelling's T², b) exceeding the 95% threshold for Q residuals, or c) exceeding the χ²-derived critical value for Mahalanobis distance. According to the Mahalanobis distance criterion, the following samples were identified as potential outliers: NT-7 in the miRNA dataset, and NT-7 and ART-3 in both the piRNA and tRNA datasets. These samples were further evaluated in combination with the additional outlier detection metrics prior to downstream analyses.
