# Supplementary material for: sncRNA signatures underlying HIV-1 restriction in elite controllers
Source: Front Cell Infect Microbiol. 2026 Oct 20;16:1926123. doi: 10.3389/fcimb.2026.1926123 (PMC13727583; doi:10.3389/fcimb.2026.1926123)
Supplement: Supplementary file 1 [file SupplementaryFile1.zip › Supplementary Figure 2.docx]

**Supplementary Figure 2.** **Interaction network of biological pathways overrepresented by GSEA.** The 50 enriched terms with the lowest False Discovery Rate (FDR) are shown. Each pathway or biological term is represented by a point. The relationship between terms was evaluated based on the genes that compose them using the Jaccard similarity index, revealing relationships with an index greater than 0.2. The size of the dots estimates the number of genes that compose them, and the FDR is shown on the color scale. The yellow arrows indicate pathways that are repressed in ECs. A. Enriched terms between EC and ART. B. Enriched terms between EC and NT.
