# Supplementary material for: sncRNA signatures underlying HIV-1 restriction in elite controllers
Source: Front Cell Infect Microbiol. 2026 Oct 20;16:1926123. doi: 10.3389/fcimb.2026.1926123 (PMC13727583; doi:10.3389/fcimb.2026.1926123)
Supplement: Supplementary file 1 [file SupplementaryFile1.zip › Supplementary Figure 3.docx]

**Supplementary Figure 3.** **Differential gene expression and overrepresentation analysis in EC.** The left plot shows a Venn diagram of differentially expressed genes (DEG) obtained in the EC vs ART and EC vs NT comparisons. The 26 DEGs collected in the intersection are represented in the adjacent heatmap. Patients were classified hierarchically using an unsupervised Euclidean approach based on the expression levels of the DEGs. The yellow and grey arrows indicate genes whose products interact physically or genetically with the TP53 protein, respectively.
