# Supplementary material for: sncRNA signatures underlying HIV-1 restriction in elite controllers
Source: Front Cell Infect Microbiol. 2026 Oct 20;16:1926123. doi: 10.3389/fcimb.2026.1926123 (PMC13727583; doi:10.3389/fcimb.2026.1926123)
Supplement: Supplementary file 1 [file SupplementaryFile1.zip › Supplementary Figure 4.docx]

**Supplementary Figure 4.** **Gene interaction network in enriched terms in the EC vs ART comparison. A.** Interaction network of enriched terms and genes. The terms are represented as colored circles according to the genes that compose them (squares). All genes represented are overexpressed in EC. **B.** The percentage of DEGs that form part of each term is represented with respect to the total number of genes that compose it. The size of the circle is related to the FDR, while the color indicates the database from which the enriched terms originate.
