# Supplementary material for: sncRNA signatures underlying HIV-1 restriction in elite controllers
Source: Front Cell Infect Microbiol. 2026 Oct 20;16:1926123. doi: 10.3389/fcimb.2026.1926123 (PMC13727583; doi:10.3389/fcimb.2026.1926123)
Supplement: Supplementary file 1 [file SupplementaryFile1.zip › Supplementary Table 1. Primers list.docx]

**Supplementary Table 1.** Primers used in the qPCR for miRNA-Target expression assessment.

| **Gene target** | **Primer sense** | **Sequence (5’-3’)** | **Tm^3^ (^o^C)** | **Amplicon size (bp^4^)** |
| --- | --- | --- | --- | --- |
| ***ACTB*** | Fw^1^ | AGGCCCAGAGCAAGAGAGGCA | 71 | 102 |
|  | Rv^2^ | CGCAGCTCATTGTAGAAGGTGTGGT | 70 |  |
| ***P53*** | Fw | GCCCCTGTCATCTTCTGTCC | 60 | 80 |
|  | Rv | TCCCAGAATGCAAGAAGCCC | 60 |  |
| ***BAK1*** | Fw | AGGTTTTCCGCAGCTACGTT | 60 | 94 |
|  | Rv | GGTAAGGTGACCATCTCTGGG | 60 |  |
| ***BCL2*** | Fw | GGTGGGGTCATGTGTGTGG | 61 | 89 |
|  | Rv | CGGTTCAGGTACTCAGTCATCC | 60 |  |
| ***CDKN1A*** | Fw | TGTCTTGTACCCTTGTGCCTC | 60 | 116 |
|  | Rv | GGAGTGGTAGAAATCTGTCATGC | 59 |  |
| ***MTOR*** | Fw | AACCTCCTCCCCTCCAATGA | 60 | 92 |
|  | Rv | TCAGCGGTAAAAGTGTCCC | 60 |  |
| ***TNPO3*** | Fw | TGACCTGTGTAGAAAAAGCAGGA | 60 | 72 |
|  | Rv | ACCAACTTCCCAAACAGCGA | 60 |  |
| ***FLNA*** | Fw | CCTGCTGGCTGTCCAGATCA | 62 | 108 |
|  | Rv | CCTGTCACGTCTGGCACGTA | 62 |  |
| ***THBS1*** | Fw | GGATCCGGCTCTGCAACTCT | 62 | 121 |
|  | Rv | CAAGGACCCCAGCCTCCATT | 62 |  |
| ***DPM2*** | Fw | CTCGTCGCCGTTAGCCTGAT | 62 | 126 |
|  | Rv | TGGGATGGCGACAGCATAGG | 62 |  |
| ***LAMP1*** | Fw | CCACCATCCAGGCGTACCTT | 62 | 70 |
|  | Rv | GGCCTGTCTTGTTCACAGCG | 62 |  |
| ***BBC3*** | Fw | ATTTGGCATGGGGTCTGCCCA | 65 | 88 |
|  | Rv | AAATCTGGCAGGGGACCCACG | 65 |  |
| ***MAFB*** | Fw | CTACGCCCAGTCTTGCAGGT | 62 | 80 |
|  | Rv | CCTGCTGAATGAGCTGCGTC | 62 |  |

1. Fw: Forward; 2. Rv: Reverse; 3. Tm: Temperature of melting; 4. bp: base pairs.
